# The Influence of Blood Contamination on Cerebrospinal Fluid Diagnostics

**DOI:** 10.3389/fneur.2019.00584

**Published:** 2019-06-12

**Authors:** Philipp Schwenkenbecher, Theda Janssen, Ulrich Wurster, Felix Franz Konen, Alexandra Neyazi, Jonas Ahlbrecht, Wolfram Puppe, Lena Bönig, Kurt-Wolfram Sühs, Martin Stangel, Tina Ganzenmueller, Thomas Skripuletz

**Affiliations:** ^1^Clinical Neuroimmunology and Neurochemistry, Department of Neurology, Hannover Medical School, Hanover, Germany; ^2^Department of Psychiatry, Social Psychiatry and Psychotherapy, Hannover Medical School, Hanover, Germany; ^3^Hannover Medical School, Institute of Virology, Hanover, Germany; ^4^Institute of Medical Virology, University Hospital Tuebingen, Tuebingen, Germany

**Keywords:** cerebrospinal fluid, blood contamination, erythrophages, siderophages, intrathecal immunoglobulin synthesis, antibody index, diagnostic pitfalls

## Abstract

**Background:** Blood contamination due to traumatic lumbar puncture presents a diagnostic pitfall in cerebrospinal fluid (CSF) analysis. It is controversially discussed if phagocytosis of erythrocytes which can be found in the CSF after subarachnoid hemorrhage can also develop *in vitro* in the presence of artificial blood contamination. Furthermore, there is no consensus about the acceptable amount of artificial blood contamination on CSF protein results.

**Methods:** Two measurement series were performed in order to investigate the role of artificial blood contamination on the possible development of erythrophages and siderophages in the CSF: (1) blood contamination was simulated *in vitro* by adding blood into the CSF. (2) CSF was investigated when blood contamination occurred during a traumatic lumbar puncture. In both types of experiments, CSF including blood was incubated for 24 h and for 72 h at room temperature or at 4°C. In the third measurement series, the effects of artificial blood contamination on CSF protein results were investigated. Blood contamination was simulated *in vitro* by adding different amounts of blood ending up with five different samples containing erythrocyte counts of 2,500, 5,000, 7,500, 10,000, and 20,000 per μl CSF.

**Results:** Cytological examination revealed no evidence of erythrophages or siderophages *in vitro*. In contrast, already a low blood contamination (2,500 erythrocytes/μl CSF) led to false pathological results of total protein and albumin. Along with increasing amounts of blood, the frequency of false pathological protein results increased. A blood contamination of 5,000 erythrocytes/μl CSF resulted in a false positive intrathecal IgM production in nearly every fifth patient. In contrast, blood contamination with 5,000 erythrocytes/μl CSF was the acceptable amount of blood which did not lead to a false positive intrathecal synthesis of IgG and IgA.

**Conclusion:** Erythrophages and siderophages do not develop *in vitro*. An extensive diagnostic work up for the source of blood in the CSF should be performed when erythrophages or siderophages are found in the CSF. The contamination of CSF with increasing volume of blood resulted in falsely elevated CSF protein concentrations. Hence, the amount of blood contamination has to be taken into consideration when interpreting CSF protein measurement results.

## Introduction

Cerebrospinal fluid (CSF) analysis is an important routine procedure if a central nervous system (CNS) infection or subarachnoid bleeding is suspected (1). In 7% of the patients with subarachnoid hemorrhage brain imaging does not detect blood, and thus, CSF analysis is required to verify the clinical diagnosis (2–6). However, artificial bleeding induced by lumbar puncture can cause peripheral blood contamination in the CSF sample leading to false-positive results (7). In order to distinguish artificial blood contamination from subarachnoid hemorrhage, counting erythrocytes in a number of consecutive tubes, centrifuging the specimen and evaluating the supernatant for xanthochromia, spectrometry for oxyhemoglobin, bilirubin determination, or ferritin measurement is performed (1, 8). However, a diagnostic gap remains (8). Cytological examination represents another important method when a subarachnoid bleeding is suspected (1). Bleedings within the CSF compartment induce activation of macrophages which phagocyte erythrocytes and then are called erythrophages (1). Erythrophages are considered to occur ~12–18 h after the bleeding event (9–11). These macrophages form haemosiderin storage after 36–48 h and are then called siderophages (10, 11). It is controversially discussed if erythrophages and siderophages exclusively occur in the CSF of patients with subarachnoid hemorrhage. It has been suggested that in some cases erythrophages and siderophages might develop *in vitro* in the CSF in the presence of artificial blood contamination and thus are not specific for subarachnoid bleeding (12). However, a recent study investigated a short-time (7 h) of incubation of CSF with blood but could not find any evidence of erythrophage development *in vitro* (13).

Another diagnostic problem is the analysis of CSF immunoglobulins (IgM, IgA, and IgG) in patients with artificially contaminated CSF. The evaluation of intrathecal immunoglobulin synthesis is the result of IgM, IgA, and IgG CSF/serum quotients to albumin CSF/serum quotients as described previously (14).

To date, there is no consensus about the acceptable amount of artificial blood contamination on CSF results. Therefore, we investigated the influence of artificial CSF contamination with different amounts of blood spiked into the CSF on the development of erythrophages and siderophages and on routine CSF results including total protein measurements, blood-CSF-barrier-function, intrathecal synthesis of immunoglobulins IgM, IgA, and IgG, and virus specific intrathecal antibody synthesis.

## Methods

### Experimental Design

Two different measurement series were performed in order to investigate the role of artificial blood contamination on the possible development of erythrophages and siderophages in the CSF. In the first experiment, blood contamination was simulated *in vitro* by adding blood from the same patient into CSF samples after routine lumbar puncture. Before the blood was added, the CSF samples were cytological controlled to be free of erythrocytes. In the second experiment, CSF samples were investigated if blood contamination occurred by a traumatic lumbar puncture. Traumatic lumbar puncture was defined by the evidence of erythrocytes in cytological examination. In both experiments, CSF including blood was incubated for 24 and 72 h at room temperature and at 4°C with the aim to imitate the standard clinical practice.

In a third measurement series, the effects of artificial blood contamination on CSF protein measurements were investigated. Blood contamination was imitated *in vitro* by adding different amounts of blood into CSF samples after routine lumbar puncture.

#### Series 1: Artificial Blood Contamination *in vitro* and Cytological Examination

In this part of the study, CSF samples contaminated with blood were generated by adding patient's EDTA blood into the CSF. Three different blood concentrations were selected: (a) 2 μl blood was incubated with 2 ml CSF (1 μl blood/1 ml CSF: mean: 3,506 ± 1,334 erythrocytes per ml CSF, range: 1,829–6,400); (b) 1 μl blood was incubated with 2 ml CSF (0.5 μl blood/1 ml CSF: mean: 2,705 ± 1,077 erythrocytes per ml CSF, range: 1,493–4,512); (c) 1 μl blood was incubated with 3 ml CSF (0.33 μl blood/1 ml CSF: mean: 1,391 ± 367 erythrocytes per ml CSF, range: 893–2,048). The mean erythrocyte count for all three concentrations was 2,541 ± 1,306 erythrocytes per ml CSF, (range 893–6,400) and 4.6 ± 0.57 million erythrocytes per μl blood (range 2.9–5.6). The CSF sample was then divided in two 1 ml samples of which one sample was stored at room temperature and the other sample was incubated at 4°C for 24 h. Cytological examination for cell type distribution with special focus on erythrophages and siderophages was then performed by two independent experienced cytologists.

These samples originated from 25 adult patients who underwent lumbar puncture for routine diagnosis. The gender distribution was 48% females to 52% males. The median age was 49 years (range 23–81). Patients were diagnosed with autoimmune diseases (*n* = 10), neurodegenerative diseases (*n* = 5), infectious diseases (*n* = 4), neoplastic diseases (*n* = 3), and idiopathic causes such as headache (*n* = 3).

In order to investigate the effects of 72 h incubation additional experiments including new CSF samples were performed. CSF samples from 11 patients were spiked with different blood concentrations in analogy to samples that were incubated for 24 h: (a) 2 μl blood was incubated with 2 ml CSF (1 μl blood/1 ml CSF: mean: 4,720 ± 2,243 erythrocytes per ml CSF, range: 2,325–9,045); (b) 1 μl blood was incubated with 2 ml CSF (0.5 μl blood/1 ml CSF: mean: 3,194 ± 1,934 erythrocytes per ml CSF, range: 1,280–7,253); (c) 1 μl blood was incubated with 3 ml CSF (0.33 μl blood/1 ml CSF: mean: 1,947 ± 1,324 erythrocytes per ml CSF, range: 576–5,120). The mean erythrocyte count for all three concentrations in CSF was 3,287 ± 2,324 (range 576–9,045) and 4.7 ± 0.90 million erythrocytes per μl blood (range 2.7–5.8). The CSF sample was then divided in two 1 ml samples of which one sample was stored at room temperature and the other sample incubated at 4°C for 72 h. The gender distribution was 45% females to 55% males. The median age was 45 years (range 24–69). Patients were diagnosed with autoimmune diseases (*n* = 6), neurodegenerative diseases (*n* = 2), infectious diseases (*n* = 2), and idiopathic causes (one patient with bell's palsy).

#### Series 2: Cytological Examination of CSF Samples With Traumatic Blood Contamination

CSF samples contaminated with blood by a traumatic lumbar puncture were used in this part of the study. A mean erythrocyte count of 3,262 ± 10,153 /μl CSF (range: 20–71,680) and 4.8 ± 0.51 million erythrocytes per μl blood (range: 3.5–5.6) was found. The CSF was again divided in two 1 ml samples of which one sample was stored at room temperature and the other sample was incubated at 4°C for 24 h. Cytological examination was then performed in analogy to the examination with artificial blood contamination *in vitro*.

This part of the study included 50 adult patients who underwent lumbar puncture for routine diagnosis. The gender distribution was 46% females and 54% males. The median age was 54 years (range 19–89). Patients were diagnosed with autoimmune diseases (*n* = 29), neurodegenerative diseases (*n* = 9), seizures (*n* = 4), an infectious disease (*n* = 1), neoplastic diseases (*n* = 2), and idiopathic causes such as headache (*n* = 5).

In order to investigate the effects of 72 h incubation additional experiments including new CSF samples were performed. CSF samples originated from 10 patients contaminated with blood by traumatic lumbar puncture were divided in two 1 ml samples and stored at room temperature and incubated at 4°C for 72 h. The mean erythrocyte count was 2,532 ± 5,750 /μl CSF (range: 56–18,787) and 4.1 ± 0.75 million erythrocytes per μl blood (range 2.6–5.0). The gender distribution was 70% females and 30% males. The median age was 64 years (range 28–83). Patients were diagnosed with neurodegenerative diseases (*n* = 4), infectious diseases (*n* = 2), idiopathic causes such as headache (*n* = 2), neoplastic diseases (*n* = 1), and autoimmune diseases (*n* = 1).

#### Series 3: The Effects of CSF Blood Contamination on CSF Protein Diagnostics

In this part of the study, CSF samples contaminated with blood were generated by adding patient's blood into the CSF samples. In order to simulate different degrees of blood contamination, each CSF sample was divided into five portions and inoculated with corresponding patient's blood to obtain samples with 2,500 erythrocytes/μl, 5,000 erythrocytes/μl, 7,500 erythrocytes/μl, 10,000 erythrocytes/μl, and 20,000 erythrocytes/μl. Each sample of CSF and serum underwent routine protein analytical procedures. The following values were investigated: total protein, albumin quotient, intrathecal synthesis of IgM, IgA, and IgG, and virus specific antibody synthesis to measles, rubella, and varicella zoster.

This part of the study comprised 25 adult patients who underwent lumbar puncture for routine diagnosis. The gender distribution was 48% females to 52% males. The median age was 67 years (range 21–89). CSF was used from patients with no clinical or CSF evidence of an inflammatory CNS disease (CSF cell count within the normal range, no evidence of an intrathecal immunoglobulin production in CSF routine diagnostics, CSF oligoclonal band negative). Patients were diagnosed with idiopathic causes such as headache (*n* = 12), neurodegenerative diseases (*n* = 10), peripheral neuropathy (*n* = 2), and a neoplastic disease (*n* = 1).

### CSF Analytical Procedures

CSF and corresponding serum samples underwent standard diagnostic procedures in the Neurochemistry Laboratory of the Department of Neurology (15). CSF leukocytes were counted manually with a Fuchs-Rosenthal counting chamber. CSF cell count ≥ 5 cells/μl was considered elevated. For cytological examinations, CSF samples were centrifuged for slide preparation. After a Pappenheim staining a combination of May-Grünwald (Merck, Darmstadt, Germany) and Giemsa staining (Sigma-Aldrich, St. Louis, USA) cells were identified under a light microscope at x250–400 magnification (16). At least 100 cells were differentiated on each slide.

Total protein in CSF was determined by a Bradford dye-binding procedure. Albumin, IgG, IgM, and IgA were measured in CSF and serum by kinetic nephelometry (Beckman Coulter IMMAGE). Age and sex-adjusted upper reference limits for total protein in CSF were used according to recent studies (17, 18). Blood-CSF barrier function was evaluated by CSF-serum albumin quotients (QAlbumin). The age-adjusted upper reference limit of QAlbumin was calculated using the formula QAlbumin = 4 + (age in years/15) which Reiber et al. suggested according to Faber et al. (19, 20). This formula is long established and evaluated for different neurological diseases (21–23). In addition, we also assessed the blood-CSF barrier function according to the more recent formula of age-adjusted upper reference limit: 8 + (age in years/25) which bases on a large cohort of control patients (17). Intrathecal synthesis of IgG, IgA, and IgM was calculated according to Reiber's revised hyperbolic function referring IgG, IgA, and IgM quotients to QAlbumin (14). CSF specific oligoclonal bands were determined by isoelectric focusing in polyacrylamide gels with consecutive silver staining (24).

Measlevirus, rubellavirus, and varicella zoster virus IgG antibody ELISAs in serum and CSF as well as subsequent calculation of the virus specific antibody index (AI) were performed at the MHH Institute of Virology on a EUROIMMUN Analyzer I using the respective Euroimmun ELISA kits. The AI was calculated according to the formula (CSF virus-IgG/serum virus-IgG)/(CSF IgG total/serum IgG total); AI values ≥ 1.5 were considered to indicate intrathecal production of virusspecific IgG (14).

### Statistical Analysis

GraphPad Prism version 5.02 was used for statistical analysis. Fisher's exact and chi-square tests were used when analyzing categorical data. Whether data were normally distributed was analyzed with D'Agostino-Pearson test. ANOVA with Bonferroni *post-hoc* test was used for group comparison and paired *t*-test for comparison of two groups. The level of statistical significance was set to 5%. Data are described by means, standard deviation, medians and ranges.

## Results

### Blood Added to CSF *in vitro* or Blood Contamination of CSF After Traumatic Lumbar Puncture Does Not Induce the Development of Erythrophages and Siderophages *in vitro*

Cytological examination results showed that the incubation of CSF samples with blood *in vitro* for 24 and 72 h did not induce the development of erythrophages or siderophages at two different storing conditions (room temperature, 4°C) (Table 1 and Supplemental Table 1). In addition, evidence of erythrophages or siderophages could not be found in CSF which was blood contaminated by traumatic lumbar puncture after 24 and 72 h *in vitro* at room temperature and 4°C (Table 2 and Supplemental Table 2). Xanthochromia did not occur after 24 and 72 h of incubation with artificial blood contamination.

**Table 1:** CSF cell distribution for CSF samples gained after artificial blood contamination incubated at room temperature and 4°C for 24 h.

**CSF cell distribution after 24 h**	**Blood added to CSF** ***in vitro***					
	**Incubated at room temperature (*****n*** **=** **25)**			**Incubated at 4**^****°****^**C (*****n*** **=** **25)**		
	**0.33 μl blood/1 ml** **CSF**	**0.5 μl blood/1 ml** **CSF**	**1 μl blood/1 ml** **CSF**	**0.33 μl blood/1 ml** **CSF**	**0.5 μl blood/1 ml** **CSF**	**1 μl blood/1 ml** **CSF**
Erythrophages	0%	0%	0%	0%	0%	0%
Lytic cells	7%	9%	6%	8%	11%	6%
Lymphocytes	64%	57%	51%	48%	41%	41%
Monocytes	6%	8%	8%	7%	11%	5%
Neutrophils	23%	26%	35%	37%	37%	48%

**Table 2:** CSF cell distribution for CSF samples gained after traumatic lumbar puncture incubated at room temperature and 4°C for 24 h.

**CSF cell distribution after 24 h**	**Blood contamination of CSF by traumatic lumbar puncture**	
	**Incubated at room temperature (*n* = 50)**	**Incubated at 4^**°**^C (*n* = 50)**
Erythrophages	0%	0%
Lytic cells	13%	15%
Lymphocytes	58%	51%
Monocytes	8%	7%
Neutrophils	21%	27%

In addition, we investigated the cell distribution in all samples after 24 and 72 h of incubation *in vitro*. The amount of lytic cells was between 6% (blood contaminated samples) and 15% (traumatic lumbar puncture at 4°C) of all cells after 24 h (*p* = 0.0046) and between 31% (blood contaminated samples) and 78% (traumatic lumbar puncture at 4°C) of all cells after 72 h (Tables 1, 2; Supplemental Tables 1, 2; *p* < 0.0001). Moreover, the frequency of lytic cells after 24 h was similar between both incubation conditions (room temperature, 4°C; *p* = 0.8480), while more cells were lytic after 72 h than after 24 h (Table 1, and Supplemental Tables 1, 2; *p* < 0.0001). The amount of lymphocytes slightly decreased, while the amount of granulocytes slightly increased after addition of increasing amounts of blood in samples after 24 h incubation time (Table 1; lymphocytes: *p* = 0.0887; granulocytes: *p* < 0.0001). After 72 h, the amount of lytic cells and granulocytes was higher in samples which were incubated at 4°C than in samples incubated at room temperature (Table 1 and Supplemental Tables 1, 2; lytic cells: *p* < 0.0001; granulocytes: *p* < 0.0001).

### The Effect of *in vitro* Blood Contamination on CSF Total Protein and Blood-CSF-Barrier Function (CSF/Serum Albumin Quotient) by Adding Blood

**Figure 1:**
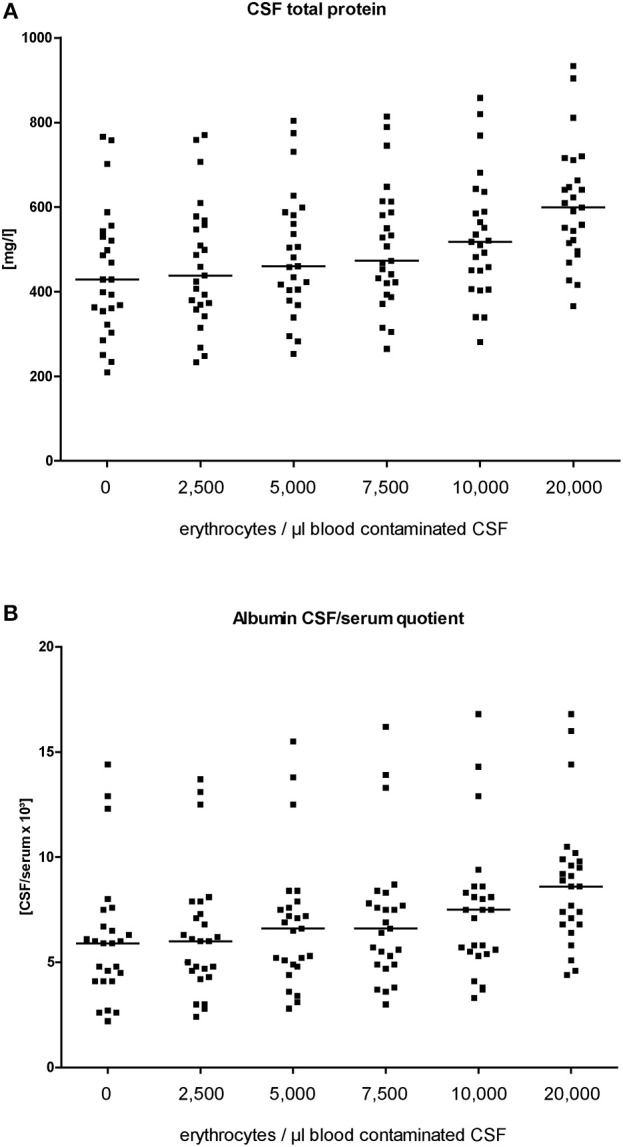
Level of CSF total protein **(A)** and albumin quotients **(B)** at each titration step (erythrocytes/μl blood contaminated CSF). Horizontal bars indicate the medians.

Five different blood volumes including erythrocyte amounts between 2,500 and 20,000 per μl CSF were investigated in order to imitate daily clinical experience. The addition of blood into the CSF continuously increased the mean level of total protein in CSF (445 ± 153 mg/l in untreated CSF and 606 ± 148 mg/l in CSF containing 20,000 erythrocytes/μl; *p* < 0.0001; Figure 1A; Table 3). Total protein was elevated in 3/25 patients (12.5%; *p* = 0.2347) before blood was added into CSF samples. The addition of blood resulted in a higher number of patients with pathological total protein results: 4/25 patients (16%; *p* = 0.1099) in CSF containing 2,500 erythrocytes/μl, 4/25 patients (16%; *p* = 0.1099) in CSF containing 5,000 erythrocytes/μl, 6/25 patients (24%; *p* = 0.0223) in CSF containing 7,500 erythrocytes/μl CSF, 8/25 patients (32%; *p* = 0.0040) in CSF containing 10,000 erythrocytes/μl CSF and 9/25 patients (36%; *p* = 0.0016) in CSF containing 20,000 erythrocytes/μl CSF.

**Table 3:** Overview of CSF protein results in response to different amounts of blood contamination.

**CSF with artificial blood containing:**	**CSF parameters**							
	**Total protein**	**QAlbumin**	**Intrathecal synthesis**			**IgG antibody index**		
			**IgG**	**IgA**	**IgM**	**Measles**	**Rubella**	**VZV**
2,500 erythrocytes/μl	**↑**	**↑** (**↑**)^*^	**–**	**–**	**–**	**–**	**–**	**–**
5,000 erythrocytes/μl	**↑**	**↑↑** (↑)^*^	**–**	**–**	**↑↑**	**–**	**–**	**–**
7,500 erythrocytes/μl	**↑↑**	**↑↑** (↑)^*^	**↑**	**↑**	**↑↑↑↑**	**–**	**–**	**–**
10,000 erythrocytes/μl	**↑↑**	**↑↑** (↑)^*^	**↑**	**↑↑**	**↑↑↑↑↑↑**	**–**	**–**	**–**
20,000 erythrocytes/μl	**↑↑↑**	**↑↑↑↑↑** (↑↑)^*^	**↑↑**	**↑↑↑↑**	**↑↑↑↑↑↑↑↑**	**–**	**–**	**–**

*The arrows indicate false positive results as compared to naive CSF without blood contamination. ↑ = 1–10% of patients with false positive results; ↑↑ = 11–20% of patients with false positive results; ↑↑↑ = 21–30% of patients with false positive results; ↑↑↑↑ = 31–40% of patients with false positive results; ↑↑↑↑↑ = 41–50% of patients with false positive results; ↑↑↑↑↑↑ = 51–60% of patients with false positive results; ↑↑↑↑↑↑↑↑ = 71–80% of patients with false positive results*.

* *↑ in brackets indicate QAlbumin above the upper reference by using the formula 8 + (age in years/25)*.

The albumin quotient (QAlbumin) is generally accepted as the best indicator to describe a blood-CSF barrier dysfunction for blood derived proteins. The mean serum albumin level was 40.1 g/l ± 5.2. Four patients had serum albumin levels between 32.6 and 34.8 g/l. Before blood was added into CSF samples, 4/25 patients (16%) showed an elevated QAlbumin indicating a blood-CSF barrier dysfunction (*p* = 0.1099; Figure 1B; Table 3). The addition of blood resulted in a higher number of patients with pathological QAlbumin values indicating a blood-CSF barrier dysfunction: 5/25 patients (20%) in CSF containing 2,500 erythrocytes/μl (*p* = 0.05), 7/25 patients (28%) in CSF containing 5,000 erythrocytes/μl (*p* = 0.0096), 7/25 patients (28%) in CSF containing 7,500 erythrocytes/μl (*p* = 0.0096), 7/25 patients (28%) in CSF containing 10,000 erythrocytes/μl (*p* = 0.0096), and 15/25 patients (60%) in CSF containing 20,000 erythrocytes/μl (*p* < 0.0001).

When using the formula 8 + (age in years/25), 3/25 patients (12%) had an elevated QAlbumin before blood was added. After addition of blood pathological QAlbumin was found in 3/25 patients (12%) in CSF containing 2,500 erythrocytes/μl (*p* = 0.2347), in 3/25 patients (12%) in CSF containing 5,000 erythrocytes/μl (*p* = 0.2347), in 3/25 patients (12%) in CSF containing 7,500 erythrocytes/μl (*p* = 0.2347), in 3/25 patients (12%) in CSF containing 10,000 erythrocytes/μl (*p* = 0.2347), and in 6/25 patients (24%) containing 20,000 erythrocytes/μl (*p* = 0.0223).

### The Effect of Blood Contamination on Intrathecal Synthesis of IgG, IgA, and IgM

**Figure 2:**
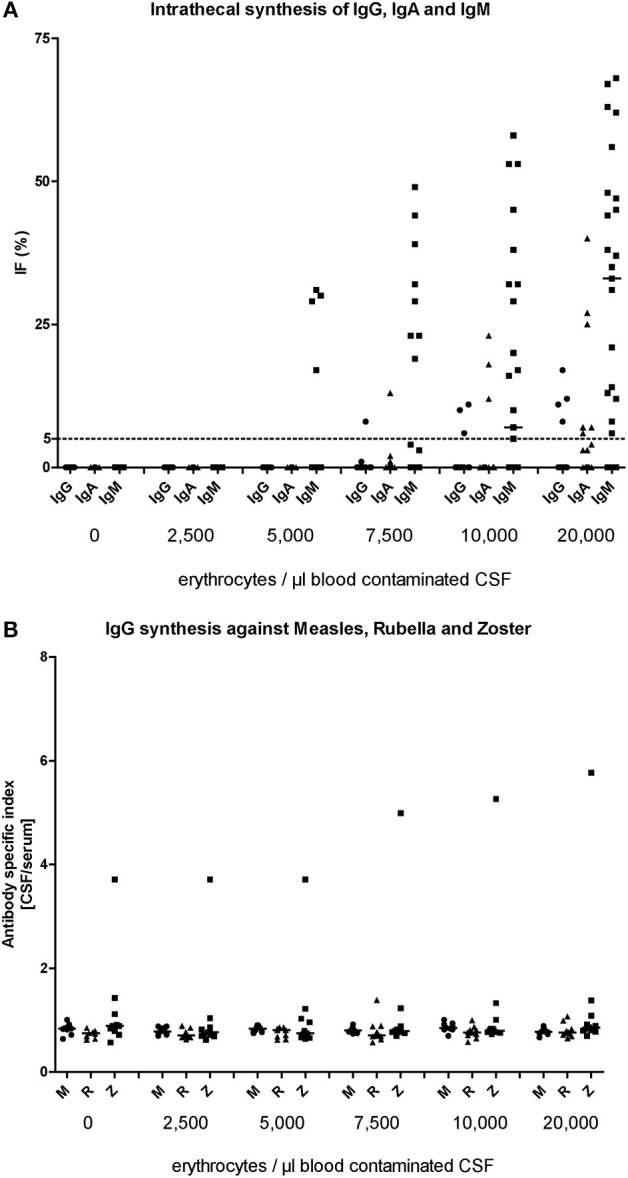
Level of artificial immunoglobulin synthesis for IgM, IgA, and IgG **(A)** and specific IgG synthesis against the viruses **M**easles, **R**ubella, and Varicella **Z**oster Virus **(B)** at each titration step (erythrocytes/μl blood contaminated CSF).

In analogy to total protein and QAlbumin, the influence of five different blood volumes (the same samples as described above) on results indicating intrathecal production of immunoglobulins as referred to Reiber-graphs, was investigated. The mean serum IgG level was 11.0 g/l ± 3.7, the IgA level was 2.5 g/l ± 1.3, and the IgM level was 1.0 g/l ± 0.5. Serum IgG concentration <7 g/dl was found in one patient, serum IgA <0.7 g/dl in three patients, and serum IgM <0.4 g/l in two patients. Before blood was added to the CSF, none of the selected patients exhibited an intrathecal synthesis of either IgG, or IgA, or IgM (Figure 2A; Table 3).

The addition of blood containing 2,500 and 5,000 erythrocytes/ μl did not result in a false positive intrathecal synthesis of IgG. A false positive intrathecal synthesis of IgG was only detected with higher blood amounts: 1/25 patient (4%) in CSF containing 7,500 erythrocytes/μl (*p* > 0.9999), 3/25 patients (12.5%) in CSF containing 10,000 erythrocytes/μl (*p* = 0.2347) and 4/25 patients (16%) in CSF containing 20,000 erythrocytes/μl (*p* = 0.1099).

Similar to IgG, a false positive intrathecal synthesis of IgA was first detected in CSF contaminated with blood containing 7,500 erythrocytes/μl (1/25 patient; *p* > 0.9999). The addition of blood containing 10,000 erythrocytes/μl resulted in 3/25 patients (12.5%; *p* = 0.2347) with a false positive intrathecal synthesis of IgA, while blood containing 20,000 erythrocytes/μl showed a false intrathecal IgA synthesis in 9/25 patients (36%; *p* = 0.0016).

For intrathecal IgM synthesis, false positive results occurred already as consequence of blood addition containing 5,000 erythrocytes/μl (4/25 patients, 16%; *p* = 0.1099) and further increased with higher blood amounts. The addition of blood containing 7,500 erythrocytes/μl resulted in 9/25 patients (36%; *p* = 0.0016) with a false positive intrathecal synthesis of IgM, while blood containing 10,000 and 20,000 erythrocytes showed a false intrathecal IgM synthesis in 14/25 patients (56%; *p* < 0.0001) and 20/25 patients (80%; *p* < 0.0001), respectively.

### The Effect of Blood Contamination on Virus Specific Intrathecal IgG Antibody Synthesis

The influence of *in vitro* blood contamination on viral specific IgG synthesis against the viruses measles, rubella, and varicella zoster was investigated in analogy to the intrathecal synthesis of immunoglobulins as described above by using the same CSF samples. Before blood was added to the CSF, only one patient exhibited an elevated antibody index against the varicella zoster virus (Figure 2B; Table 3). The addition of blood containing 2,500, 5,000, 7,500, 10,000, and 20,000 erythrocytes/μl did not result in a false positive antibody index for any virus. The mean antibody index before blood was added was 0.8 ± 0.1 for measles virus, 0.7 ± 0.1 rubella virus and 1.2 ± 0.9 for varicella zoster virus. In the last titration step with 20,000 erythrocytes/μl the mean antibody index was 0.8 ± 0.1 for measles virus, 0.8 ± 0.1 rubella virus and 1.3 ± 1.5 for varicella zoster virus. The differences did not reach significant level.

## Discussion

In the present study we show that artificial blood contamination of the CSF did not induce the development of erythrophages and siderophages *in vitro* after 24 and 72 h of incubation. In contrast, the artificial contamination of CSF with blood resulted in false pathological CSF protein results and the proportion of false results continuously increased with increasing blood.

CT scan of the head is able to uncover a subarachnoid bleeding with a high sensitivity of approximately 86–93% 6 h after manifestation (1, 25). However, the sensitivity decreases to approximately 50% already after 1 week of bleeding occurrence (1, 25). Thus, in patients with suspected subarachnoid hemorrhage but normal CT of the head the diagnostic algorithm recommends a diagnostic lumbar puncture to detect subarachnoid hemorrhage 8–12 h after onset of the headache (9). However, diagnostic doubt arises when trauma induced by the needle causes artificial contamination of CSF with blood (2–4, 26). In such cases, the cytological examination represents a routine diagnostic approach to uncover erythrophages, which develop 12–18 h after contact with blood (9). After additional 36–48 h erythrophages produce haemosiderin deposits and are then called siderophages (1). Siderophages do not play a critical role in the first 3 days after bleeding, but the sensitivity and specificity increase during the progress and siderophages may even be detected several months after an insult (1, 10, 11). To date, it is controversially discussed if erythrophages and siderophages are exclusively generated in the CSF of patients with subarachnoid bleeding. It has been reported that blood contamination of the CSF as consequence of a previous lumbar puncture might induce the development of siderophages (27). Furthermore, it has been supposed that in some cases erythrophages might develop *in vitro* in the CSF in the presence of artificial blood contamination being not specific for a subarachnoid bleeding (12). Here we have shown that erythrophages and siderophages do not develop *in vitro* after blood contamination and an incubation time of 24 and 72 h. Our results are in line with the results of Dersch and colleagues who have recently reported that erythrophages do not develop *in vitro* after 7 h of incubation (13). The presence of xanthochromia in CSF is considered to be another indicator for subarachnoid bleeding in patients with negative CT scan (10, 28–30). In analogy to the absence of erythrophages and siderophages we did not detected visually xanthromic CSF in our experiments. We thus suggest that erythrophages and siderophages only develop *in vivo* in the CSF compartment as response to blood. The sensitivity of the human color vision is considered to be not sufficient to detect slight yellowish tint due to small amount of bilirubin (5, 31). In a previous study including patients with subarachnoid hemorrhage, visual inspection of CSF supernatant, revealed that only 47% of patients had xanthromic CSF while CSF of 53% patients was colorless (32). In a study using artificially lysed erythrocytes in distilled water the sensitivity of visual xanthromia was even 26.6% (5). The findings of these studies suggest that the lysis rate of erythrocytes *in vivo* might be higher. On the other side, the lack of xanthromia in our study might be explained by small amounts of erythrocytes, while true subarachnoid hemorrhages tend to have higher erythrocyte counts (26).

Artificial blood contamination of CSF samples presents not only a dilemma for the diagnosis of a subarachnoid bleeding but also influences CSF protein analysis leading to false pathological results (4). Our results show that already a low blood contamination (containing 2,500 erythrocytes/μl CSF) induced elevated levels above the cut-off of 500 mg/l of total protein and age related albumin quotients indicating a disturbed blood-CSF-barrier function. With increasing amounts of blood contamination (up to 20,000 erythrocytes/μl), the frequency of false pathological total protein and albumin quotient values increased up to every second patient of our study population.

Another diagnostic problem is the analysis of immunoglobulins (IgM, IgA, and IgG) in the CSF artificially contaminated with blood. The concentrations of immunoglobulins are magnificent higher in serum than in CSF and thus blood contamination may pretend to a false positive evidence of an intrathecal production of immunoglobulins indicating inflammatory processes in the CSF. Due to the different molecular weight of immunoglobulins (IgM>IgA>IgG) only very low concentrations of IgM are usually found in CSF. Blood contamination of the CSF will therefore affect the above proteins exactly in the same order with falsified IgM in the first place. The influence of blood contamination on CSF results should therefore be higher for IgM, followed by IgA and IgG. Our results are in line with these assumptions and conclusively show that false positive production of IgM was predominant in our experiments, followed by IgA, and IgG. A blood contamination of 5,000 erythrocytes/μl CSF resulted in a false positive intrathecal IgM production in nearly every fifth patient. In contrast, blood contamination with 5,000 erythrocytes/μl CSF did not induce a false positive intrathecal production of IgG and IgA. An artificial intrathecal synthesis of IgG and IgA occurred in every tenth patient after addition of blood containing 7,500 erythrocytes/μl CSF. We thus suggest a cut off of 5,000 erythrocytes/μl CSF for analyses of intrathecal IgG synthesis. In the case of a contaminated CSF with blood a falsely elevated intrathecal IgG synthesis will reliably be detected by the absence of oligoclonal bands in the CSF. This qualitative method to detect intrathecal IgG synthesis is less susceptible for blood contamination.

Interestingly, CSF contamination with even higher amounts of blood (up to 20,000 erythrocytes/μl CSF) did not lead to false positive IgG antibody index against viruses. The cut-off of blood contamination for analyses of the IgG antibody index against viruses might be therefore higher than 20,000 erythrocytes/μl CSF. However, since blood contamination increased the mean IgG antibody index against rubella and measles for 0.1, false positive results could be the consequence in cases of borderline IgG antibody indices.

In conclusion, erythrophages and siderophages did not develop *in vitro* and we thus suggest an extensive diagnostic work up for the source of blood when erythrophages and siderophages are found in the CSF. The contamination of CSF with blood resulted in false pathological CSF protein results in some patients. Contamination of the CSF with 5,000 erythrocytes/μl was the acceptable amount of blood which did not induce a false positive intrathecal synthesis of IgG.

## Data Availability

Data supporting the findings can be found in the tables. Additional data extracted may be shared upon request.

## Ethics Statement

The study was approved by the Ethic Committee of the Hannover Medical School, Carl-Neuberg-Str.1, 30625 Hannover (1 December 2012; 1322-2012). Only participants older than 18 years were included. All participants of this study gave written informed consent. The study is in accordance with the Declaration of Helsinki regarding ethical conduct of research involving human subjects.

## Author Contributions

PS: participated in the design of the study, collected and analyzed the data and drafted the manuscript. TJ: collected the data, analyzed the data and drafted the manuscript. UW was responsible for CSF analysis, analyzed the data and contributed in drafting the manuscript. FK: collected and analyzed the data. AN, JA, WP, LB and KS contributed in drafting the manuscript. MS and TG analyzed the data and contributed in drafting the manuscript. TS: conceived the study, analyzed the data and drafted the manuscript. All authors read and approved the final manuscript.

### Conflict of Interest Statement

The authors declare that the research was conducted in the absence of any commercial or financial relationships that could be construed as a potential conflict of interest.

## Supplementary Material

Supplemental Table 1 CSF cell distribution for CSF samples gained after artificial blood contamination incubated at room temperature and 24°C for 72 h.

Supplemental Table 2 CSF cell distribution for CSF samples gained after traumatic lumbar puncture incubated at room temperature and 4°C for 72 h.

## Abbreviations

CSF: cerebrospinal fluid
CNS: central nervous system
OCB: CSF-specific oligoclonal bands
Alb: CSF-serum albumin quotients
AI: antibody index.
